# Sarcomatoid mesothelioma: unusual findings and literature review

**DOI:** 10.1093/jscr/rjac512

**Published:** 2022-11-19

**Authors:** Brittni Clopton, Winnie Long, Monica Santos, Armand Asarian, Romulo Genato, Philip Xiao

**Affiliations:** American University of the Caribbean School of Medicine, Cupecoy, St. Maarten; Department of Surgery, the Brooklyn Hospital Center, Icahn School of Medicine, Brooklyn, NY, USA; Department of Surgery, the Brooklyn Hospital Center, Icahn School of Medicine, Brooklyn, NY, USA; Department of Surgery, the Brooklyn Hospital Center, Icahn School of Medicine, Brooklyn, NY, USA; Department of Surgery, the Brooklyn Hospital Center, Icahn School of Medicine, Brooklyn, NY, USA; Department of Pathology, the Brooklyn Hospital Center, Icahn School of Medicine, Brooklyn, NY, USA

## Abstract

Sarcomatoid mesothelioma is an aggressive disease secondary to its propensity to undergo rapid growth, show inconsistent expression of tumor markers and invade surrounding tissues. Therefore, there are numerous obstacles that clinical researchers face as they look for new methods to diagnose and treat the malignancy. We present a case of sarcomatoid mesothelioma, originally thought to be metastasis from renal cell carcinoma.

## INTRODUCTION

Mesothelioma is a pleural malignancy that originates from pleural mesothelial cells, which are cells that detect and react to noxious stimuli and are found as a mono layer lining the pleura. This cancer has been associated with long-term exposure to the naturally occurring mineral fiber, asbestos. Early symptoms are often nonspecific and may be initially diagnosed as pneumonia. Associated tumor markers may not be consistently expressed and the malignancy often progresses rapidly. These features often lead to delays in diagnosis and poor prognosis.

The main pathological morphologies of mesothelioma include non-epithelioid, epithelioid and sarcomatoid. The epithelioid type offers the best prognosis, followed by non-epithelioid and sarcomatoid. Most mesotheliomas can be diagnosed with biopsy and immunohistochemical staining, though the sarcomatoid type has been shown to have the most inconsistent expression of two common tumor markers: cytokeratin and calretinin.

## CASE PRESENTATION

We present a case of a 74-year-old male with a past medical history of hypertension, type II diabetes mellitus, alpha thalassemia trait, monoclonal gammopathy, chronic lymphocytic leukemia and a recently diagnosed left kidney mass and left pleural effusion. Patient presented to the emergency department with a worsening cough, occasional sputum production and shortness of breath with exertion for 2 weeks. Pertinent positive symptoms included back pain, headaches and chills. On exam, the patient was hemodynamically stable with decreased breath sounds in bilateral lower segments and mild left upper quadrant abdominal tenderness.

Chest X-ray demonstrated a heterogeneous 12.8 × 9.6 × 8.1 cm mass suspicious for neoplasm in the left lower lobe, with malignant pleural thickening/modularity encasing the entire left hemithorax circumferentially. This measured up to 3.5 cm at its widest diameter in the left lung base. Other noted findings included three pulmonary nodules suspicious for metastasis noted in the right lung with the largest measuring 1.0 cm, and a previously known 9.0 × 7.6 cm heterogeneous soft tissue mass arising from the upper pole of the left kidney, most compatible with neoplasm.

Prior to this encounter, the left kidney mass was previously recognized to be causing mild-to-moderate left hydronephrosis and flattening of the left renal vein. At the time this mass was hypothesized to be renal cell carcinoma (RCC) versus lymphoma. Patient had not yet undergone nephrectomy and positron emission tomography–computed tomography. IR-guided biopsy of the left lung mass was consistent with malignant sarcomatoid mesothelioma.

**Figure 1:**
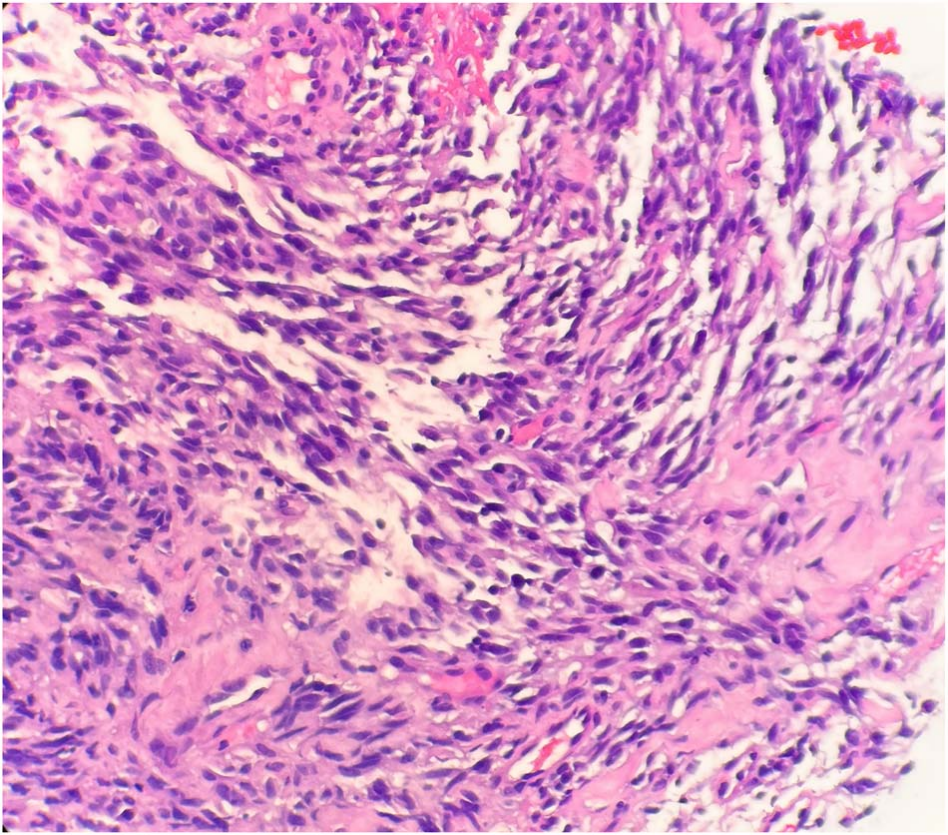
Microscopic examination reveals spindle tumor cells in sclerotic and myxoid stroma (HE stain x40).

**Figure 2:**
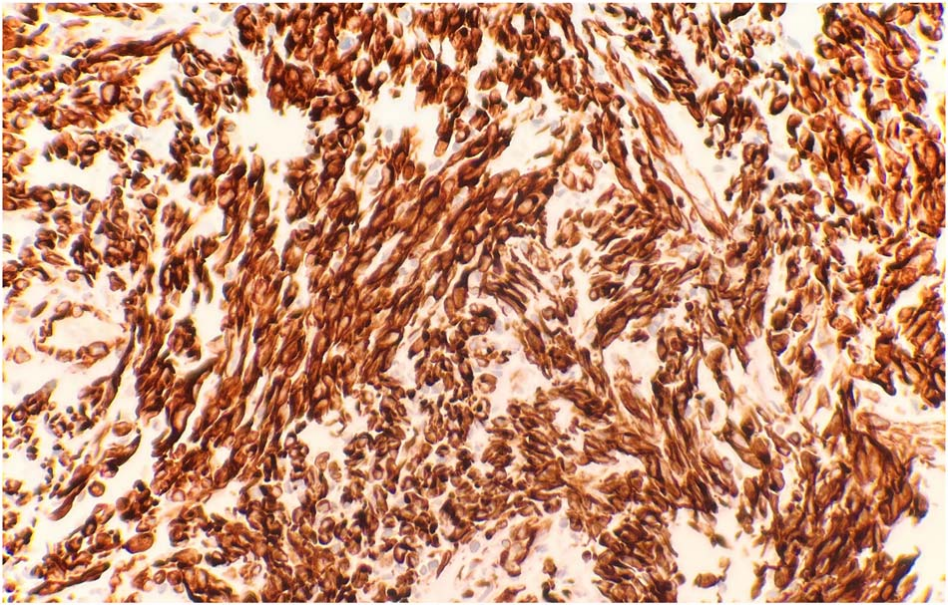
Immunohistochemical stain reveals that tumor cells are positive for WT-1 (IHC stain x40).

The pathological sections taken from the mass demonstrated spindle-tumor cells within a focal sclerotic and myxoid stroma with increased mitotic activity; no necrosis was visualized (Fig. 1). The vessels from the pleural mass showed a staghorn-like appearance. Tumor cells biopsied from the lung mass were positive for vimentin and CD10, as well as biomarkers for sarcomatoid mesothelioma: WT1 and D2–40 (Fig. 2). The tissue sample was negative for immunostains keratin and calretinin, which is unusual for mesothelioma. The lung mass biopsy was also negative for cytokeratin (CK) and PAX8, which ruled out the possibility of metastatic RCC. Due to the visualization of staghorn-appearing vessels, malignant solitary fibrous mesenchymal tumor was considered as well but was ruled out due to lack of STAT6 expression.

## DISCUSSION

Sarcomatoid mesothelioma is regarded as the ``least common, but most aggressive of the three major histological types of mesotheliomas''. This aggressiveness is due to its poor response rate to methods of treatment such as chemotherapy and surgical removal, with the average survival rate being 5–6 months across several studies [1].

As reflected in this case, sarcomatoid mesothelioma has inconsistent expression of the common tumor markers used in immunohistochemistry [2]. Usually, the staining qualities for this neoplasm are positive for CK and calretinin. However, the negative expressions for keratin and calretinin, as seen in this patient, occurs in <5% of cases for sarcomatoid mesothelioma [3, 4]. The prognosis of sarcomatoid mesothelioma has been found to be the worst out of the three types of mesothelioma [5]. Therefore, treatment and therapy options are limited to improving quality of life and prolonging survival time. There has been no definitive cure found for mesothelioma.

Surgery has been found to improve visible tumor burden, relieve dyspnea and reduce the occurrence of pleural effusions. Extrapleural pneumonectomy (EPP) and pleurectomy/decortication (P/D) are the most common operative techniques used [6]. However, these operations come with risks of their own. EPP has been practiced in the context of clinical trials and requires ‘complete resection of the affected visceral pleural and parietal pleura, the lungs, the diaphragm and even part of the pericardium [6]. This full resection leads to high perioperative mortality and morbidity rates, reaching nearly 32% in the past. With adjustments to surgical technique, morbidity and mortality rates can now be as low as 4%. P/D requires complete resection of the visceral and parietal pleura, and retention of the lung [5]’; as such, the trauma burden on the patient is relative, with comparatively lower perioperative mortality and morbidity rates at 1.5–5.4% [6].

Chemotherapy has been shown to be effective as both neoadjuvant and adjuvant therapy in the treatment of sarcomatoid mesothelioma [5]. The treatments approved by the US Food and Drug Administration and the European Union for the management of mesothelioma include pemetrexed in conjunction with cisplatin, both of which are now considered first line chemotherapeutic options [7].

Trimodality therapy (TMT) is a potential curative treatment option involving EPP with neoadjuvant or adjuvant chemotherapy and adjuvant radiotherapy [8]. A systematic review conducted by Cao *et al*. [8] compared outcomes of TMT studies with the Mesothelioma and Radical Surgery (MARS) trials. MARS trials consisted of three cycles of platinum-based chemotherapy, radical surgery by EPP and radical radiotherapy [9]. It was found in an intention-to-treat study for TMT consisting of neoadjuvant gemcitabine and cisplatin, followed by EPP offered a median survival of 23 months compared with an average of 14.4 months in the MARS trials [9]. These results are encouraging; however, further evidence is needed to determine the efficacy of both TMT and MARS trials for long-term survival due to inconsistencies in literature.

Molecular targeted therapy and immunotherapy are also being explored as future treatment options. A study conducted by Wu *et al*. [10] explored the incidence of a higher T-regulatory cell count in patients diagnosed with mesothelioma. These high T-Reg counts have been seen to inhibit the normal function of T-Reg cells and downregulate the secretion of interleukin-10 (IL-10) and transforming growth factor-b (TGF-b). Additionally, CTLA-4/CD152, a leukocyte differentiation antigen, was found to potentially have anti-neoplastic effects by regulating the immune response [1, 10].

## CONCLUSION

Our patient presented with a pleural lung mass which was initially thought to be a metastasis from RCC but was a biopsy-proven sarcomatoid mesothelioma. Sarcomatoid mesothelioma has the worst prognosis due to the inconsistent expression of common tumor markers, and rapid progression. Current practicing therapies are aimed at combining surgery, neoadjuvant/adjuvant chemotherapy as well as neoadjuvant radiotherapy to improve survival rate and improve symptoms. There are many randomized controlled trials, prospective studies and intention-to-treat studies that are evaluating the efficacy of therapies such as TMT and MARS trials. Molecular targeted therapy and immunotherapy have also made promising contributions toward the future of management for mesothelioma and will need to be studied further. All the aforementioned therapies have been shown to have an important role in the treatment of MPM going forward.
